# Feeding Practices in Very Preterm and Very Low Birth Weight Infants in an Area Where a Network of Human Milk Banks Is in Place

**DOI:** 10.3389/fped.2018.00387

**Published:** 2018-12-06

**Authors:** Elettra Berti, Monia Puglia, Silvia Perugi, Luigi Gagliardi, Cristiana Bosi, Anna Ingargiola, Letizia Magi, Elena Martelli, Simone Pratesi, Emilio Sigali, Barbara Tomasini, Franca Rusconi

**Affiliations:** ^1^Anna Meyer Children's University Hospital, Florence, Italy; ^2^Health Agency of Tuscany, Florence, Italy; ^3^Careggi University Hospital, Florence, Italy; ^4^Versilia Hospital, Viareggio, Italy; ^5^San Giovanni di Dio Hospital, Florence, Italy; ^6^San Donato Hospital, Arezzo, Italy; ^7^Santo Stefano Hospital, Prato, Italy; ^8^University Hospital of Pisa, Pisa, Italy; ^9^University Hospital of Siena, Siena, Italy

**Keywords:** donor milk, human milk, mother's own milk, complementary milk, full enteral feeding, preterm, very low birth weight

## Abstract

**Background:** Great variability in enteral feeding practices for very preterm (<32 weeks gestational age-GA) and very low birth weight infants (VLBW; ≤1,500 g) have been reported. We aimed to describe data on enteral feeding in Tuscany (Italy), where a network of 6 donor milk banks is in place.

**Methods:** A 4-years (2012–2015) observational study was performed analyzing the database “TIN Toscane online” on very preterm and VLBW infants. The database covers all 25 hospitals with a neonatal unit.

**Results:** Data concerning the beginning of enteral nutrition were available for 1,302 newborns with a mean (standard deviation) GA of 29.3 (2.9) weeks, while information at the time of full enteral nutrition was available for 1,235 and at discharge for 1,140. Most infants (74.1%) started enteral feeding during the first 24 h of life. Overall, 80.1% of newborns were fed exclusive human milk, donor milk having the larger prevalence of use (66.8%). Few infants (13.3%) started with exclusive mother's milk. Full enteral feeding was achieved using exclusive human milk in most cases (80%). Full enteral feeding was reached earlier in newborns who were fed human milk than in those fed formula, regardless of GA. Sixty-four percent of infants were still fed with any human milk at discharge. When data at the achievement of full enteral nutrition and at discharge were analyzed stratified by the type of milk used to start enteral feeding, newborns initially fed donor milk presented the highest prevalence (91.3%) of exclusive human milk at full enteral feeding, an important period to prevent necrotizing enterocolitis, while no differences were observed at discharge.

**Conclusions:** Donor milk was widely used for newborns during the first hours of life, when mother's milk availability may be quite challenging. Starting enteral nutrition with donor milk was associated with early start of enteral feeding and early achievement of full enteral nutrition without affecting mother lactation. The overall prevalence of human milk at discharge (when donor milk is not available anymore) was high (64%), irrespective of the type of milk used to start nutrition.

## Introduction

Perinatal interventions and care practices have improved survival and long-term outcomes for very preterm (<32 weeks of gestation) and very low birth weight (VLBW: birth weight ≤1,500 g) infants over the last two decades (1, 2). Nutrition is a major element of care for these infants; nevertheless, great variability in enteral feeding practice (3–5) has been reported. A debate is still underway in the scientific community on the best feeding strategy, including time of initiation and the rate of incrementing feeding. Mother's own milk is recommended as the best feeding for all newborns, including preterm and VLBW infants, for its multiple short- and long-term health benefits (6–9). Donor human milk represents the best alternative whenever breastfeeding is impossible, or mother's own milk is unavailable, as commonly occurs in neonatal intensive care units (NICUs) in the very 1st days of infant life (6–9).

To date, only few studies have reported feeding practices adopted for very preterm and VLBW infants during their hospitalization, and little is known on the use of donor human milk in NICUs.

A very recent observational study in 162 Neonatal Units in England showed a low prevalence of use of donor milk in newborn infants <32 weeks gestation, with wide variations (2–61%) across networks. Networks without donor milk availability possibly choose to wait for mother's milk rather than administer formula milk because of the increased perceived risk of NEC; this resulted in newborns fed at a later postnatal age (10). On the other hand, a large study conducted by the Center for Disease Control and Prevention in the US reported that the percentage of neonatal care facilities using donor milk increased 74% between 2011 and 2015. Donor human milk use was more likely in facilities with higher breast-feeding rates and in a state with a milk bank (11).

The aim of the present study is to describe feeding practices currently adopted for very preterm and VLBW infants born in Tuscany, a region of central Italy where a network of donor milk banks is in place and where there is an area-based, web-based registry (TIN Toscane on-line).

We describe feeding practices during NICU hospitalization, focusing on the prevalence of use of mother's own milk, donor human milk and formula feeding at three different time points of enteral nutrition: the beginning of enteral feeding, the achievement of full enteral nutrition, and discharge.

## Methods

A 4 years (2012–2015) observational study was performed based on “TIN Toscane online,” an official web-based registry of the Tuscany Regional Health Service. Since 2009, the registry covers all 25 hospitals with delivery units and NICUs in the region and prospectively collects information on maternal and neonatal characteristics of all infants under 32 weeks of gestational age (GA) or with a birth weight under 1,500 g. Since 2012, data concerning feeding practices have also been registered. Written consent for the data collection was obtained from parents. In each unit, a doctor or a nurse directly accesses the registry using a personal ID and password and completes standardized structured forms for each newborn who meets inclusion criteria. Personal health information collected in the registry are de-identified.

Data are collected from neonatal admission until discharge or death, and include demographic characteristics of the mother, obstetric history, and information on delivery, clinical characteristics of the newborn at birth and during the hospital stay, as well as medical or surgical treatments received. Data quality is checked centrally every 2–3 months. Details about perinatal organization in Tuscany and “TIN Toscane online” are reported elsewhere (12).

Types of feeding included mother's own milk, either suckled directly at the breast or freshly expressed and administered by bottle, syringe, or naso/orogastric tube; donor human milk, and formula. For this study, we define as exclusive human milk either exclusive mother's milk, or donor milk, or a mix of the two regardless of the addition of milk's fortifier; “any human milk” refers to the use of human milk (mother's own milk or donor milk) alone or combined with formula; complementary milk is human milk (donor and/or mother's own milk) plus formula.

Feeding practices during NICU hospitalization were analyzed at three different time points: at the beginning of enteral feeding, including minimal enteral feeding; at the achievement of full enteral nutrition—defined as the total daily intake requirement administered via enteral nutrition only, without any parenteral nutrition—and at discharge home.

## Results

A total of 1,398 infants were included in the registry in the study period. Their main characteristics are summarized in Table 1. Congenital anomalies were considered present if the infant had a major birth defect (from a pre-specified list) recorded.

**Table 1:** Principal characteristics of the 1,398 newborn infants <32 weeks gestation or <1,500 g included in the registry from 1-01-2012 to 31-12-2015.

**Population characteristics**	***n* (%) or mean ±*SD***
Gestational age < 32 weeks 22–25 weeks 26–28 weeks 29–31 weeks	1,142 (81.7) 186 (13.3) 258 (18.5) 698 (49.9)
Birth weight < 1,500 g and gestational age ≥32 weeks	256 (18.3)
Gestational age (weeks)	29.3 ± 2.9
Weight (grams)	1,204.5 ± 373.5
Males	717 (51.3)
Singleton	863 (61.8)
Vaginal delivery	293 (21.0)
Antenatal steroids	1,179 (84.6)
Apgar score V minute	7.9 ± 1.6
Ventilation and/or oxygen support	1,266 (91.1)
Patent ductus arteriosus	538 (38.9)
Bacterial late onset sepsis	55 (4.0)
Major birth defects	56 (4.0)
Deaths	157 (11.2)
Necrotizing enterocolitis	40 (2.9)
Surgery for necrotizing enterocolitis	36 (2.6)
Focal gastrointestinal perforation	35 (2.5)

### Beginning of Enteral Nutrition

**Figure 1:**
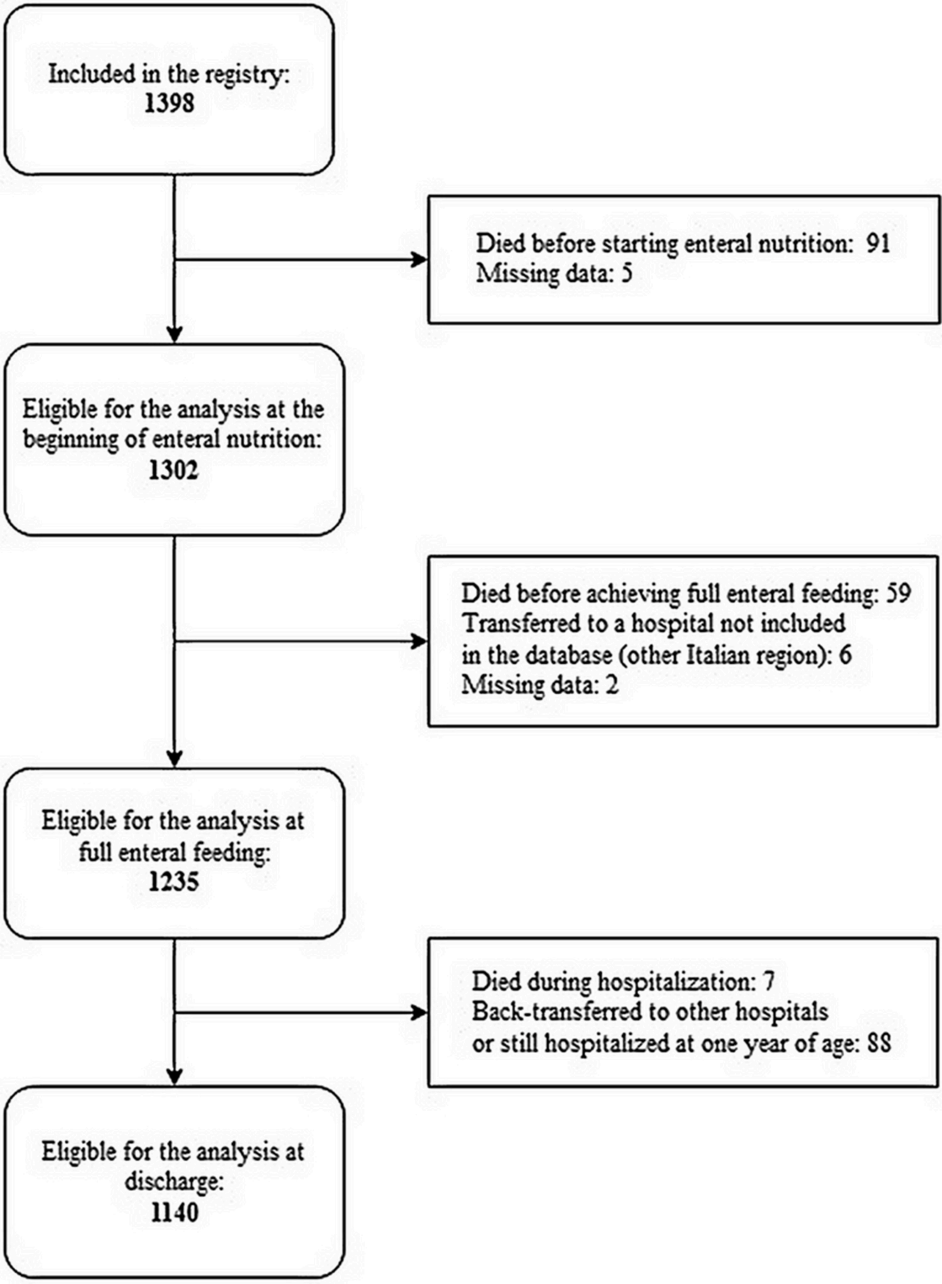
Study population.

Information concerning the beginning of enteral nutrition were available for 1,302 cases of 1,398 born in the study period (Figure 1).

**Figure 2:**
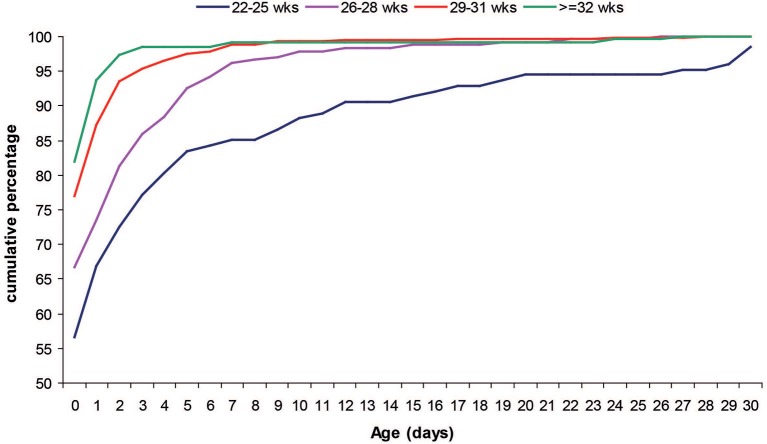
Postnatal age at the beginning of enteral feeding by GA classes.

Most of the infants (74.1%) started enteral feeding during the first 24 h of life. Infants with a lower GA started enteral feeding later than peers with higher GA (Figure 2). Mean (SD) age at start of enteral nutrition was 3.7 (7.7) days in infants younger than 25 weeks GA, 1.5 (3.3) days in infants 26–28 weeks GA, 0.6 (2.1) days in infants 29–31 weeks GA and 0.5 (2.4) in those >31 weeks GA and <1,500 g.

Human milk—more frequently, donor milk—was widely used to start enteral nutrition (80.9% of cases) (Table 2). In infants who received donor milk, enteral nutrition started very early, at a mean age of 0.4 ± 1.6 days.

**Table 2:** Feeding type at different times during hospitalization.

**Feeding type**	**At the beginning of enteral nutrition**		**At full enteral feeding**		**At discharge**	
	***n***	**%**	***n***	**%**	***n***	**%**
Mother's own milk	173	13.3	426	34.5	319	28.0
Donor milk	870	66.8	308	24.9	–	–
Mother's own milk + donor milk	–	–	255	20.6	–	–
Complementary milk	10	0.8	147	12.0	411	36.0
Formula	249	19.1	99	8.0	410	36.0
Total	1,302		1,235		1,140	

Two-hundred fifty nine infants were fed formula or complementary milk at the beginning of enteral nutrition, and most of them (86.9%) were born in the same NICU. The remaining infants initially fed with formula were distributed in 7 centres.

### Full Enteral Feeding

Data on feeding at the time of full enteral nutrition were available for 1,235 newborns (Figure 1). Full enteral feeding was achieved using any human milk in most cases (92%), and mother's own milk had the highest prevalence of use (Table 2).

The median age at the achievement of full enteral nutrition was 11 days (interquartile range-IQR: 7–20 days). Full enteral feeding time was reached earlier in newborns who were fed human milk than in those fed formula, regardless of GA. Mean time ± SD to full enteral feeding was 34.7 ± 14.2 vs. 54.5 ± 47.2 days in infants 22–25 weeks GA; 20.4 ± 10.0 vs. 40.2 ± 32.7 days in those 26–28 weeks; and 10.9 ± 8.3 vs. 18.8 ± 15.3 days in those 29–31 weeks GA; all *P* < 0.001). When feeding type at time of full enteral nutrition was stratified by the type of milk at the start of enteral feeding, newborns who were fed donor milk at the beginning presented a very high prevalence (91.3%) of exclusive human milk at full enteral feeding (Table 3).

**Table 3:** Type of milk at full enteral feeding stratified by type of milk at start of enteral nutrition.

	**Milk at full enteral nutrition**				
**Milk at the start of enteral nutrition**	**Mother's own milk**	**Donor milk**	**Mother's own milk and donor milk**	**Formula**	**Complementary milk**
Mother's own milk	57 35.2%	2 1.2%	28 17.3%	20 12.3%	55 34.0%
Donor milk	273 32.9%	279 33.6%	206 24.8%	18 2.2%	54 6.5%
Complementary milk	0	0	0	2 22.2%	7 77.8%
Formula	94 40.7%	26 11.3%	21 9.1%	59 25.5%	31 13.4%

*P < 0.001 (Pearson chi-square)*.

### Feeding at Discharge Home

The median age at discharge home was 49 days (IQR: 36–71). Feeding data at discharge home were available for 1,140 infants (Table 2). Sixty four percent of infants were still fed any human milk at discharge. Extremely preterm newborns below 26 weeks of GA presented a higher prevalence of use of formula (70.2%) compared to infants with higher GA (42.5% at 26–28 weeks; 31.18% at 29–31 weeks; 30.4% at ≥32 weeks) (*P* < 0.001). The type of milk at the start of enteral nutrition did not influence the type of feeding at discharge (Table 4).

**Table 4:** Type of milk at discharge home by type of milk at the start of enteral nutrition.

	**Milk at discharge**		
**Milk at the start of enteral nutrition**	**Mother's own milk**	**Complementary milk**	**Formula**
Mother's own milk	41 25.8%	54 34.0%	64 40.2%
Donor milk	224 28.5%	296 37.6%	267 33.9%
Complementary milk	2 22.2%	4 44.5%	3 33.3%
Formula	51 28.0%	57 31.3%	74 40.7%

*P = 0.503 (Pearson chi-square)*.

## Discussion

The present study gives a picture of enteral feeding practices for very preterm and VLBW infants in Tuscany, a region of central Italy where a network of six donor human milk banks is in place in order to ensure donor human milk provision to all the NICUs in the Region. In our population, the early introduction of enteral feeding was largely performed using exclusive human milk, and primarily donor milk. Full enteral feeding was achieved using exclusive human milk in more than 90% of cases and among these mother's own milk had the highest prevalence of use. Full enteral feeding was reached earlier in newborns fed exclusive human milk, and infants initially fed donor milk presented the highest prevalence of exclusive human milk at full enteral feeding. The type of milk at start of enteral nutrition did not influence that at discharge home.

Donor milk is often necessary in very premature infants to start enteral feeding during the first 24–48 h of life, since mother's own milk availability may be quite challenging in this period. In infants who received donor milk, enteral nutrition started very early. The availability of donor milk allowed the double benefit of safe early enteral feeding, particularly in the lower GA classes, and of a full enteral feeding with any human milk in almost all newborns (92%), mostly (80%) with exclusive human milk. Most newborns were fed exclusive human milk for at least 11 days of life, and this aspect is noteworthy, especially for extremely low birth weight infants in order to prevent late-onset sepsis and necrotizing enterocolitis. For these outcomes several authors have reported a protection when human milk is introduced up to >50 ml/kg/day (13–18). Several positive aspects are in fact associated to a fast achievement of full enteral feeding with human milk: bioactive (anti-infective, growth factors) substances in human milk, better digestibility, and shorter exposure to central vascular lines (6, 18–20). On the other hand, a recent double-blind randomized control trial found no significant effect of pasteurized donor milk during the first 10 days of life in preventing serious infections and NEC in VLBW infants when donor milk was used as a supplemental feeding whenever own mother's milk was insufficiently available during the first 10 days of life (21).

Despite the large number of newborn infants who started enteral nutrition with human milk, a consistent proportion (19.9%) was fed milk formula or complementary milk. This practice was almost entirely attributable to a single NICU with no milk bank in the hospital and that must therefore refer to the neighboring banks. In this NICU the use of formula to start enteral nutrition has been gradually decreasing over the study period, possibly related to the implementation of the milk banks' network and the improvement of donor milk availability in the Region. The characteristics (e.g., gestational age, birth weight) of neonates fed formula were not different from those of neonates who were fed human milk. A large observational study in England showed that a wide variation of donor milk use among networks could not be explained by differences in patients characteristics or presence of human milk bank within the network. According to the authors this indicates uncertainty about optimal clinical practice in relation to the use of pasteurized donor milk (10) and the need to have randomized controlled trials on this topic.

Interestingly, having started enteral feeding with donor milk did not adversely affect the proportion of infants fed mother's own milk when full enteral feeding was achieved. This confirms that a breastfeeding support in very preterm infants is part of a wider culture within the neonatal intensive care units and the early use of bank human milk is an integral part of this culture (22, 23).

The overall prevalence of mother's milk was quite elevated even at discharge, although the discontinuation of donor milk before discharge led to an increase of formula fed infants. Proportion of infants fed mother's own milk at discharge (27%) is superimposable to that reported in a previous investigation in neonatal intensive care units in Italy (24).

## Conclusions

This study confirms the importance of having an organized network of human milk banks throughout the territory to start enteral feeding early in very preterm infants and thus allowing a rapid achievement of full enteral feeding safely, particularly in the lower GA groups.

It also demonstrates the need to implement more effective strategies from the moment of achieving full enteral feeding to discharge to support the use of mother's own milk and succeed in obtaining better rates of breastfeeding at discharge.

The Tuscany region has established the Regional Network of Donor Human Milk Banks in 2008 (ReBLUD) and is actively engaged in the promotion of the culture of donor human milk, indicating the adequate availability of donor milk as an element of essential strategic value to support the care of critical newborn infants.

## Author Contributions

FR, EB, MP, SP, and LG: Conception and design of the study; CB, AI, LM, EM, SP, ES, and BT: Collection of data and critical revision of the manuscript; FR, EB, and MP: Analysis and interpretation of the data; FR, EB, SP, and LG: Drafting of the manuscript and critical revision.

### Conflict of Interest Statement

The authors declare that the research was conducted in the absence of any commercial or financial relationships that could be construed as a potential conflict of interest.
